# The metabolic effects of resumption of a high fat diet after weight loss are sex dependent in mice

**DOI:** 10.1038/s41598-023-40514-w

**Published:** 2023-08-14

**Authors:** Santiago Guerra-Cantera, Laura M. Frago, María Jiménez-Hernaiz, Roberto Collado-Pérez, Sandra Canelles, Purificación Ros, Jorge García-Piqueras, Iris Pérez-Nadador, Vicente Barrios, Jesús Argente, Julie A. Chowen

**Affiliations:** 1 Department of Endocrinology, Hospital Infantil Universitario Niño Jesús, Instituto de Investigación La Princesa, Madrid, Spain; 2 Department of Pediatrics, Universidad Autónoma de Madrid, Madrid, Spain; 3 Centro de Investigación Biomédica en Red de Fisiopatología de la Obesidad y Nutrición (CIBEROBN), Instituto de Salud Carlos III, Madrid, Spain; 4 Department of Endocrinology, Hospital Universitario Puerta de Hierro-Majadahonda, Madrid, Spain; 5 IMDEA Food Institute, CEI UAM + CSIC, Madrid, Spain

**Keywords:** Physiology, Endocrinology

## Abstract

Dietary restriction is a frequent strategy for weight loss, but adherence is difficult and returning to poor dietary habits can result in more weight gain than that previously lost. How weight loss due to unrestricted intake of a healthy diet affects the response to resumption of poor dietary habits is less studied. Moreover, whether this response differs between the sexes and if the insulin-like growth factor (IGF) system, sex dependent and involved in metabolic control, participates is unknown. Mice received rodent chow (6% Kcal from fat) or a high-fat diet (HFD, 62% Kcal from fat) for 4 months, chow for 3 months plus 1 month of HFD, or HFD for 2 months, chow for 1 month then HFD for 1 month. Males and females gained weight on HFD and lost weight when returned to chow at different rates (p < 0.001), but weight gain after resumption of HFD intake was not affected by previous weight loss in either sex. Glucose metabolism was more affected by HFD, as well as the re-exposure to HFD after weight loss, in males. This was associated with increases in hypothalamic mRNA levels of IGF2 (p < 0.01) and IGF binding protein (IGFBP) 2 (p < 0.05), factors involved in glucose metabolism, again only in males. Likewise, IGF2 increased IGFBP2 mRNA levels only in hypothalamic astrocytes from males (p < 0.05). In conclusion, the metabolic responses to dietary changes were less severe and more delayed in females and the IGF system might be involved in some of the sex specific observations.

## Introduction

Obesity is in part due to poor dietary habits, including increased consumption of diets rich in fat. Caloric restriction is one of the most frequent methods for weight loss; however, there is often a “rebound effect”, or an increased weight gain once unhealthy dietary habits are resumed^1,2^. This weight gain may be due to impairment of the neural circuitry that controls metabolism and body weight^3^. Other factors reported to contribute to this phenomenon include changes in adipocyte metabolism^4^, alterations in gut hormone levels, and a decrease in the resting energy expenditure rate or modifications in the reward system^5^. Improved dietary habits that include exclusion of foods rich in saturated fats and an increased intake of healthy foods, for example the Mediterranean diet, have been shown to improve weight loss and overall health even better than restrictive diets^6,7^. However, how weight loss due to this type of regimen affects the metabolic response to resumption of poor dietary habits is not known.

The insulin-like growth factor (IGF) system participates in a myriad of functions in the organism^8–11^, including metabolism^12–14^ as the two ligands of this system, IGF1 and IGF2, are anabolic hormones^15^ due to their structural similarity with insulin and sharing its hypoglycemic effects^16^. The main source of circulating IGFs is the liver^17^, and although they can cross the blood–brain barrier^18^, they are also produced centrally by neurons^19,20^, astrocytes^21^ and other glial cells^21,22^. Likewise, the six IGF-binding proteins (IGFBPs) that bind IGFs modifying their half-live, distribution and functions^23^ are also produced centrally, with IGFBP2 being one of the most prominently produced.

Sex differences in the IGF system^24–26^ are involved in the differences in growth^27^, and possibly metabolic responses, of males and females. In both humans and mice, females appear to be more protected against diet-induced obesity and its comorbidities than males, at least during younger ages^28–30^, with sex steroids participating in this protection^31–33^. Nutritional status alters the circulating IGF system in humans^34–36^ and in mice both systemically and centrally^37^ in a time- and sex-dependent manner, with some parameters normalizing after weight loss in children with obesity^38^. We recently reported that after 8 weeks on a high fat diet (HFD) circulating IGF2 levels are higher in mice of both sexes^37^, but after 12 weeks they remained elevated only in females, whereas IGFBP2 levels were decreased in males^39^. Hypothalamic IGF2 expression is also regulated by diet^37,40^ and both IGF2 and IGFBP2 are reportedly involved in glucose metabolism^41–43^, as is central^44^ and peripheral^45,46^ IGF1. Thus, the IGF system could participate in the differences between males and females in their responses to dietary change and weight gain.

The hypothalamus is responsible for the integration of metabolic signals and the regulation of feeding behavior^47,48^, with astrocytes in this brain area actively participating in metabolic control mechanisms^49^, including glucose and lipid sensing^50^. Moreover, astrocytes are an important source of IGF1 in the brain^21,22,51^; thus, part of their metabolic actions could involve production of IGFs and possibly other members of this family such as IGFBP2.

The aims of this study were to evaluate the potential sex differences, both centrally and peripherally, in the metabolic susceptibility to resumption of a HFD after weight loss in mice and the possible implications of the IGF system. We hypothesized that male and female mice have different metabolic responses to dietary changes, with the central and circulating IGF systems also being differentially regulated.

## Results

### Body weight and fat mass

**Figure 1:**
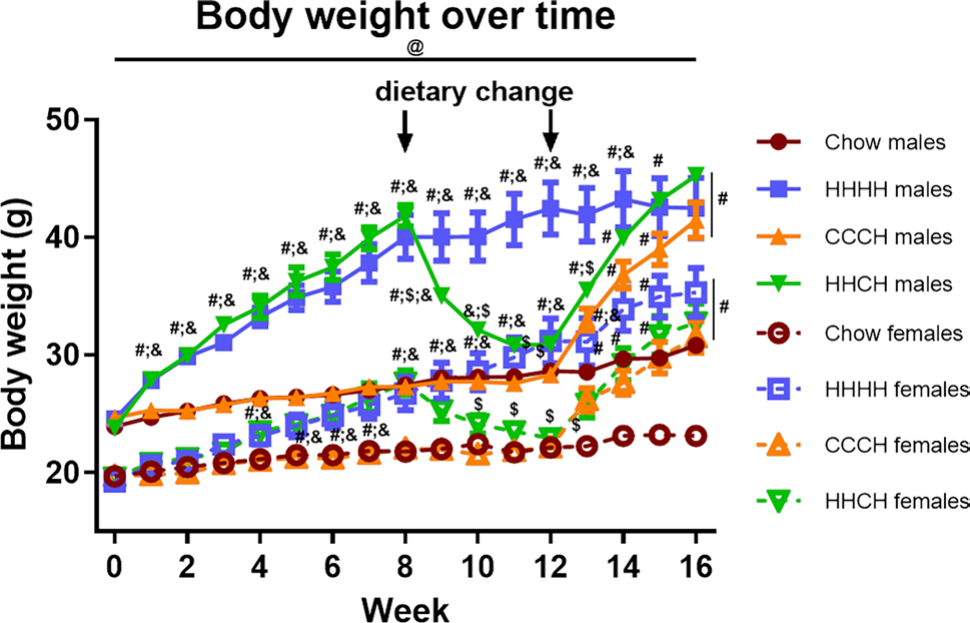
Body weight throughout the study in male and female mice exposed to chow, a high-fat diet (HFD) for 4 months (HHHH), a chow diet for 3 months and an additional month on HFD (CCCH), or HFD for 2 months followed by chow for 1 month and then HFD for the last month (HHCH). #, Different from chow in the same sex; $, different from HFD in the same sex; &, different from CCCH in the same sex; @, different between sexes on the same diet. n = 9.

The significant results of the statistical analysis of body weight are shown in Supplementary Table 1. Body weight throughout the study (Fig. 1) changed over time and was modulated by sex and the diet, with an interaction between these factors.

Body weight was greater in males than females at all study points regardless of dietary regimen. Body weight was affected by sex and diet from week 1 to week 12. All groups on the HFD weighed more than those on chow, with this reaching significance in males from week 1 (F_(3,35)_ = 10.5, p < 0.001) onward and in females after 4 weeks (F_(3,35)_ = 5.4, p < 0.01).When HHCH mice were switched from HFD to chow, males required one week (F_(3,34)_ = 32.7, p < 0.001) and females two weeks (F_(3,35)_ = 11.6, p < 0.001) for those switched to chow to weigh less than those continuing on HFD. Males also responded more rapidly to the re-exposition to HFD in the HHCH group than females, requiring one week (F_(3,34)_ = 20.1, p < 0.001) to achieve significant weight gain, while females required 2 weeks in the HHCH (F_(3,35)_ = 12.4, p < 0.001) and 3 weeks in CCCH (F_(3,35)_ = 12.6, p < 0.001) groups. Moreover, HHCH mice reached the body weight of the HHHH mice at week 14, whereas CCCH mice reached them at week 15 in both males and females.

**Figure 2:**
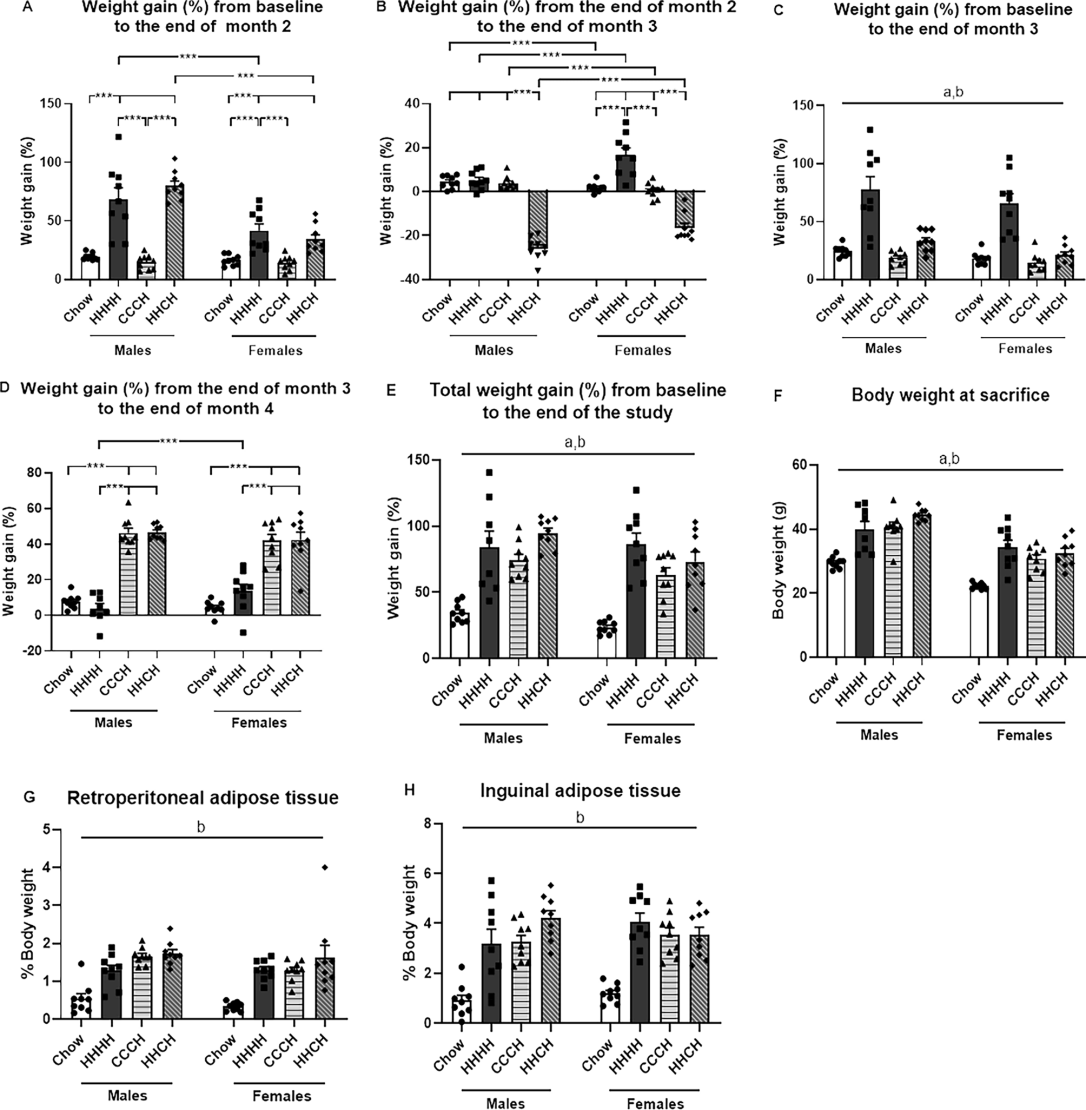
Percent weight gain compared to baseline to the end of month 2 (**A**), weight gain from the end of month 2 to the end of month 3 (**B**), weight gain from baseline to the end of month 3 (**C**), weight gain from the end of month 3 to the end of month 4 (**D**), percent total weight gain from baseline to the end of the study (**E**) and final body weight (**F**), as well as the percentage of retroperitoneal adipose tissue (**G**) and inguinal adipose tissue (**H**) in male and female mice fed with chow or a high-fat diet (HFD) for 4 months (HHHH), a chow diet for 3 months and an additional month on a HFD (CCCH), or HFD for 2 months followed by chow for 1 month and then HFD for the last month (HHCH). ***p < 0.001. a: effect of the sex, b: effect of the diet. n = 9.

The significant results of the statistical analysis of weight gain at each point of dietary change and final fat mass are shown in Supplementary Table 2. The percent change in body weight from baseline to the end of month 2 (Fig. 2A) was influenced by sex and diet, with an interaction between these factors. The HFD induced weight gain in both males (F_(3,35)_ = 37.1, p < 0.001) and females (F_(3,35)_ = 13.1, p < 0.001), but this weight gain was greater in males (p < 0.001). The percent weight gain from the end of month 2 to the end of month 3 (Fig. 2B) was influenced by sex and diet, with an interaction between these factors. HHCH mice of both sexes lost weight on the chow diet (M: F_(3,35)_ = 135.9, p < 0.001, F: F_(3,35)_ = 46.2, p < 0.001) and this decrease was greater in males than females (F_(1,17)_ = 12.3, p < 0.01). From the end of month 2 to the end of month 3, HHHH females continued to gain more weight than chow mice, while HHHH males did not (Fig. 2C). During the last month of the study, both sex and diet affected weight gain. The percent weight gain from the end of month 3 to the end of the month 4 (Fig. 2D) did not differ between CCCH and HHCH mice, although they gained relatively more weight than the chow and HHHH mice (males: F_(3,34)_ = 117.8, p < 0.001; females: F_(3,35)_ = 32.4, p < 0.001). During the last month of the study, HHHH females gained relatively more weight than males of the same group.

Total weight gain relative to baseline (Fig. 2E) and final body weight (Fig. 2F) were dependent on sex, being higher in males than females, and diet, with an overall increase in the HHHH, CCCH and HHCH groups, with no apparent differences between the regimens of HFD intake.

The relative amounts of retroperitoneal adipose tissue (Fig. 2G) and inguinal adipose tissue (Fig. 2H) were similarly affected by the dietary regimens, with an overall increase in all groups that consumed HFD.

### Energy intake

**Figure 3:**
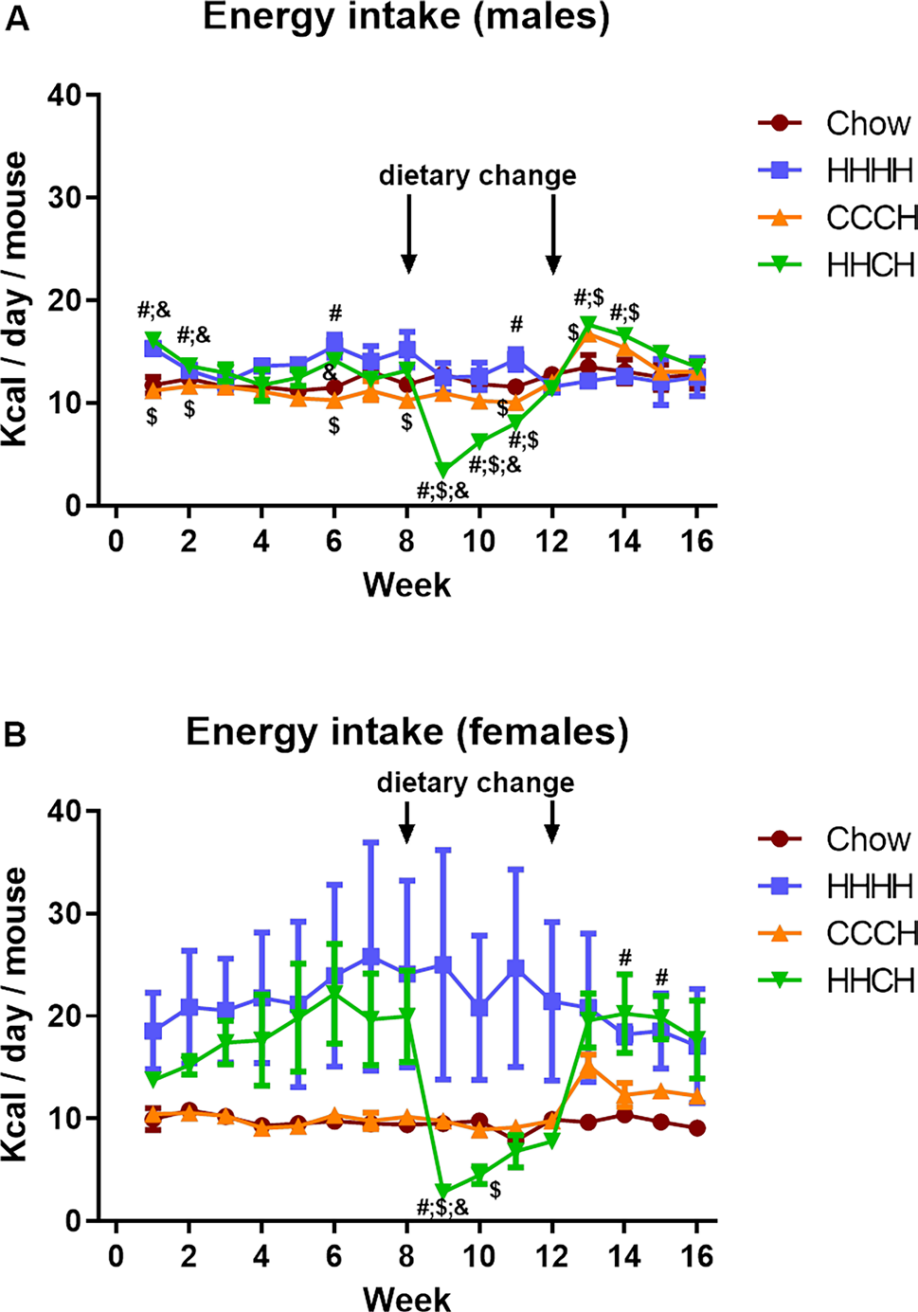
Weekly energy intake throughout the study in male (**A**) and female (**B**) mice ingesting chow or a high-fat diet (HFD) for 4 months (HHHH), chow for 3 months and an additional month on HFD (CCCH), or HFD for 2 months followed by chow for 1 month and then HFD for the last month (HHCH). #, Different from chow in the same sex; $, different from HFD in the same sex; &, different from CCCH in the same sex. n = 3 (number of cages per group).

The significant results of the statistical analysis of weekly energy intake are shown in Supplementary Table 3 and although the statistical analysis was done simultaneously on all groups, for clarity data for males (Fig. 3A) and females (Fig. 3B) are represented separately.

Energy intake was altered by time and diet from week 1 to week 15, with an interaction between time and diet. No effect of sex was found, possibly due to the high variability in female groups consuming HFD. When males were first exposed to the HFD there was an increase in energy intake (F_(3,11)_ = 28.4, p < 0.001 and F_(3,11)_ = 24.6, p < 0.001) that returned to control levels after two weeks and then fluctuated throughout the study. When HHCH mice returned to a chow diet, energy intake was rapidly reduced (F_(3,11)_ = 14.4, p = 0.001) and then returned to control levels. When HHCH mice were again exposed to a HFD at the end of month 3, there was no difference in their energy intake from the CCCH group that received HFD for the first time. In females, when HHCH returned to a chow diet they also rapidly reduced energy intake (F_(3,11)_ = 4.2, p < 0.05) that normalized during the following month. The increase in energy intake during the last month of the study did not differ between the HHCH and CCCH groups.

The global energy intake parameters are shown in Table 1. There was no difference in the total number of Kcal consumed per mouse. When energy consumption was adjusted for body weight, females consumed more Kcal than males, with no dietary effects. From baseline to the end of month 2, energy intake was higher in mice consuming HFD, while from the end of month 2 to the end of month 3 the mice switched from HFD to chow ingested fewer Kcal compared to the rest of the groups. From the end of month 3 to the end of month 4 there was an overall increase in the CCCH and HHCH groups. Mean energy efficiency (during the entire study), represented as the weight gained in grams per calories consumed, was higher in males and increased in all groups that consumed HFD in both sexes.

**Table 1:** Energy intake in male (M) and female (F) mice fed with chow or a high-fat diet (HHHH) for 4 months, chow diet for 3 months and an additional month on a HFD (CCCH), or HFD for 2 months followed by chow for 1 month and then HFD for the last month (HHCH).

	Chow M	HHHH M	CCCH M	HHCH M	Chow F	HHHH F	CCCH F	HHCH F	Sig
Total Kcal/mouse/day	12.2 ± 0.4	13.3 ± 0.8	11.8 ± 0.1	12.4 ± 0.2	9.7 ± 0.1	21.3 ± 6.8	10.6 ± 0.3	15.3 ± 2.1	NS
Total Kcal/mouse/day/100 g	44.5 ± 0.8	37.0 ± 3.9	40.5 ± 0.4	35.0 ± 0.3	44.3 ± 0.6	81.5 ± 28.0	46.2 ± 0.8	60.4 ± 6.8	a, p < 0.05
Kcal/mouse/day from baseline to the end of month 2	11.9 ± 0.4	14.1 ± 0.8	11.0 ± 0.2	13.3 ± 0.5	9.8 ± 0.1	22.1 ± 7.1	10.0 ± 0.3	18.2 ± 3.2	b, p < 0.05
Kcal/mouse/day from the end of month 2 to the end of month 3	12.3 ± 0.4	12.7 ± 0.9	10.8 ± 0.2	7.3 ± 0.2	9.3 ± 0.2	23.0 ± 8.9	9.4 ± 0.4	5.5 ± 0.7	b, p < 0.05
Kcal/mouse/day from baseline to the end of month 3	12.0 ± 0.3	13.6 ± 0.8	10.9 ± 0.2	11.3 ± 0.3	9.6 ± 0.1	22.4 ± 7.7	9.8 ± 0.3	14.0 ± 1.9	b, p < 0.05
Kcal/mouse/day from the end of month 3 to the end of month 4	13.0 ± 1.2	12.3 ± 0.9	14.6 ± 0.3	15.6 ± 0.1	9.7 ± 0.1	17.9 ± 4.2	13.1 ± 0.2	19.3 ± 3.0	b, p < 0.05
Total energy efficiency	4.5 × 10^–3^ ± 0.0	9.2 × 10^–3^ ± 0.0	12.2 × 10^–3^ ± 0.0	12.5 × 10^–3^ ± 0.0	3.0 × 10^–3^ ± 0.0	7.3 × 10^–3^ ± 0.0	9.8 × 10^–3^ ± 0.0	7.1 × 10^–3^ ± 0.0	a, p < 0.01 b, p < 0.001

a: effect of the sex, b: effect of the diet

*NS* not significant. n = 3 (number of cages per group).

### Glucose metabolism

**Figure 4:**
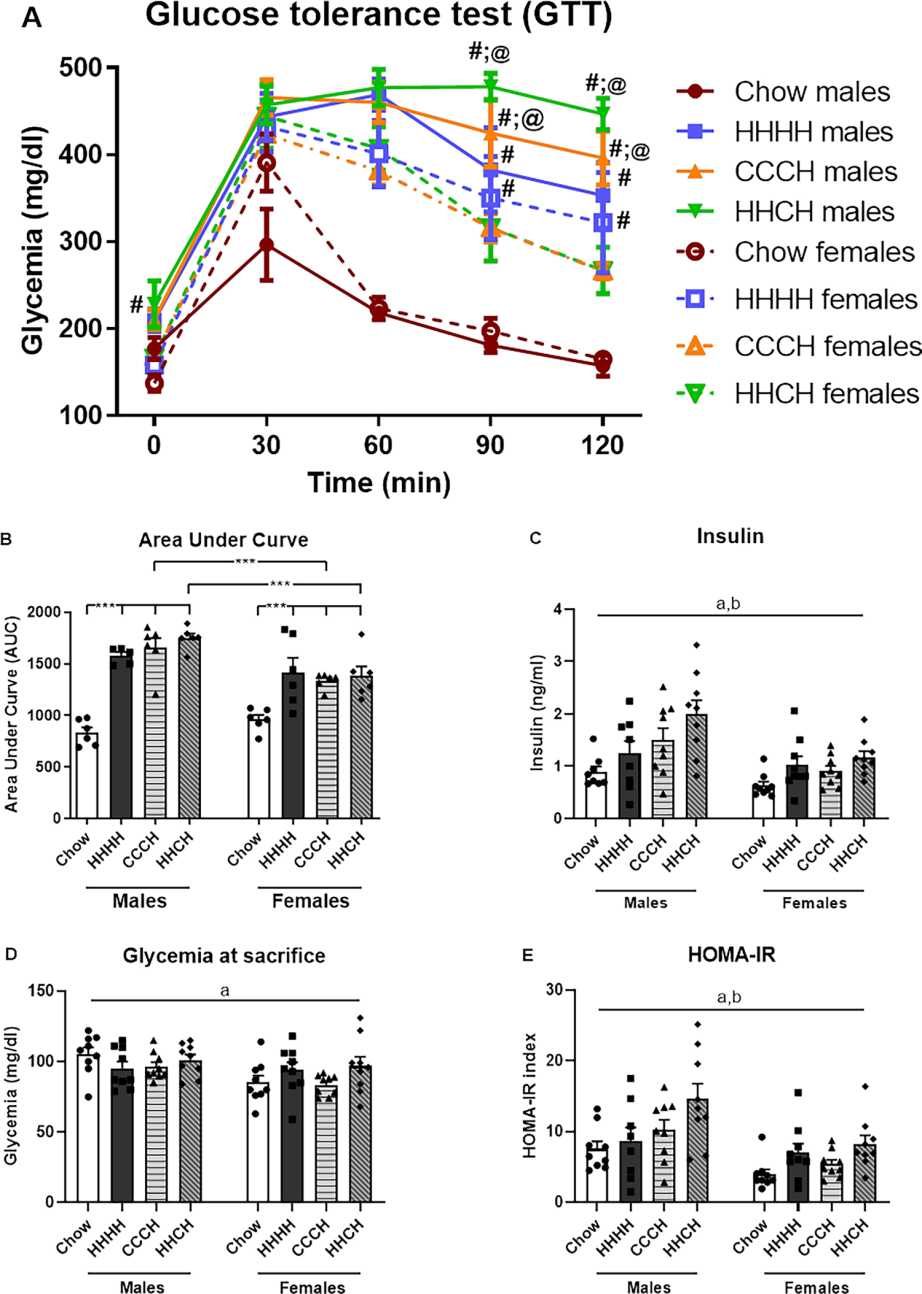
Glycemia during the glucose tolerance test (GTT; **A**), the area under the curve (AUC; **B**) in the GTT, peripheral insulin levels (**C**) and glycemia at sacrifice (**D**) and Homeostatic Model Assessment for Insulin Resistance (HOMA-IR; **E**) in male and female mice fed with chow or a high-fat diet (HFD) for 4 months (HHHH), chow diet for 3 months and an additional month on a HFD (CCCH), or HFD for 2 months followed by chow for 1 month and then HFD for the last month (HHCH). ***p < 0.001; a: effect of the sex, b: effect of the diet. #, Different from chow; @, differences between sexes on the same diet. n = 6.

Time affected glycemia levels during the GTT (Fig. 4A), with interactions between time and sex, time and diet, sex and diet, and time, sex and diet (see Supplementary Table 4 for statistical analysis).

Consuming HFD, regardless of the regimen, affected glycemia in a sex and time-dependent manner. Basal glycemia was higher in CCCH and HHCH mice compared to the chow group in both sexes (M: F_(3,22)_ = 8.4, p = 0.001; F: F_(3,23)_ = 4.0, p < 0.05), whereas in HHHH mice it was only elevated in males. Glycemia was higher in male HHHH (F_(1,10)_ = 16.2, p < 0.01), CCCH (F_(1,11)_ = 10.5, p < 0.01) and HHCH (F_(1,11)_ = 5.0, p < 0.05) compared to females of the same groups. At 30 min all groups had an increase in glycemia, whereas at 60 min glycemia returned to baseline in chow fed mice, remaining elevated in the HHHH, CCCH and HHCH groups, with this elevation being higher in males than females. At 90 min glycemia was elevated in HHHH, CCCH and HHCH males (F_(3,22)_ = 18.9, p < 0.001), whereas it was only elevated in HHHH females (F_(3,23)_ = 4.2, p < 0.05). At this time glycemia was higher in CCCH (F_(1,11)_ = 6.7, p < 0.05) and HHCH (F_(1,11)_ = 15.3, p < 0.01) males compared to females of the same groups. At 120 min glycemia remained elevated in HHHH, CCCH and HHCH males (F_(3,22)_ = 25.4, p < 0.001), whereas in females it was only elevated in the HHHH group (F_(3,23)_ = 4.1, p < 0.05), with CCCH and HHCH males continuing to have higher glycemia than females on the same diet regimen (F_(1,11)_ = 16.0, p < 0.01 and F_(1,11)_ = 31.3, p < 0.001, respectively). Mice in the HHHH, CCCH or HHCH groups had a higher AUC (Fig. 4B) than those consuming chow in both males (F_(3,22)_ = 46.5, p < 0.001) and females (F_(3,23)_ = 6.1, p < 0.01). Males had a higher AUC than females on the CCCH (F_(1,11)_ = 10.0, p = 0.01) and HHCH (F_(1,11)_ = 13.3, p < 0.01) dietary regimens.

At the end of the study, circulating insulin levels (Fig. 4C) were higher in males than females, and increased by HFD intake regardless of regimen. Glycemia levels at sacrifice (Fig. 4D) and the HOMA index (Fig. 4E), which was increased by HFD consumption, were also higher in males than females.

### Plasma levels of leptin and members of the IGF system

**Figure 5:**
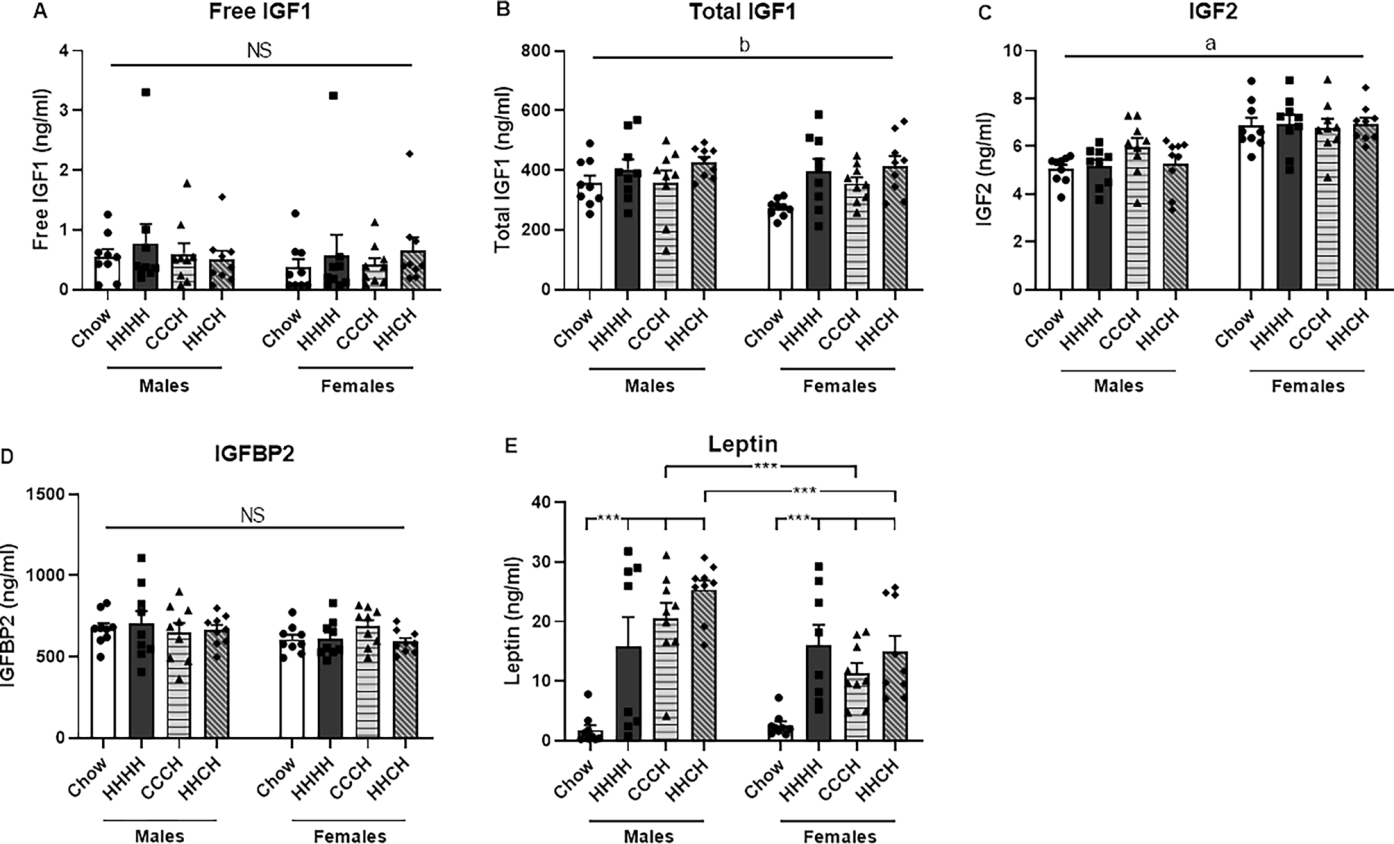
Circulating levels of free insulin-like growth factor (IGF) 1 (**A**), total IGF1 (**B**), IGF2 (**C**), IGF binding protein (IGFBP) 2 (**D**) and leptin (**E**) in mice fed chow or a high-fat diet (HFD) for 4 months (HHHH), chow diet for 3 months and an additional month on a HFD (CCCH), or HFD for 2 months followed by chow for 1 month and then HFD for the last month (HHCH). ***p < 0.001. a: effect of the sex, b: effect of the diet. *NS* non-significant. n = 9.

Significant results of the statistical analysis of circulating levels of members of the IGF system and leptin are shown in Supplementary Table 5. Circulating free IGF1 levels (Fig. 5A) were not altered by sex or diet, while there was an overall effect of HFD to increase total IGF1 levels (Fig. 5B). Females had higher circulating IGF2 levels (Fig. 5C) than males, with no effect of diet. IGFBP2 levels (Fig. 5D) were unaffected by sex or diet. Plasma leptin levels (Fig. 5E) were increased by HFD intake (M: F_(3,34)_ = 13.8, p < 0.001; F: F_(3,34)_ = 7.4, p = 0.001) with males having higher levels than females in CCCH (F_(1,17)_ = 8.9, p < 0.01) and HHCH (F_(1,17)_ = 11.5, p < 0.01) mice.

### The hypothalamic IGF system

**Figure 6:**
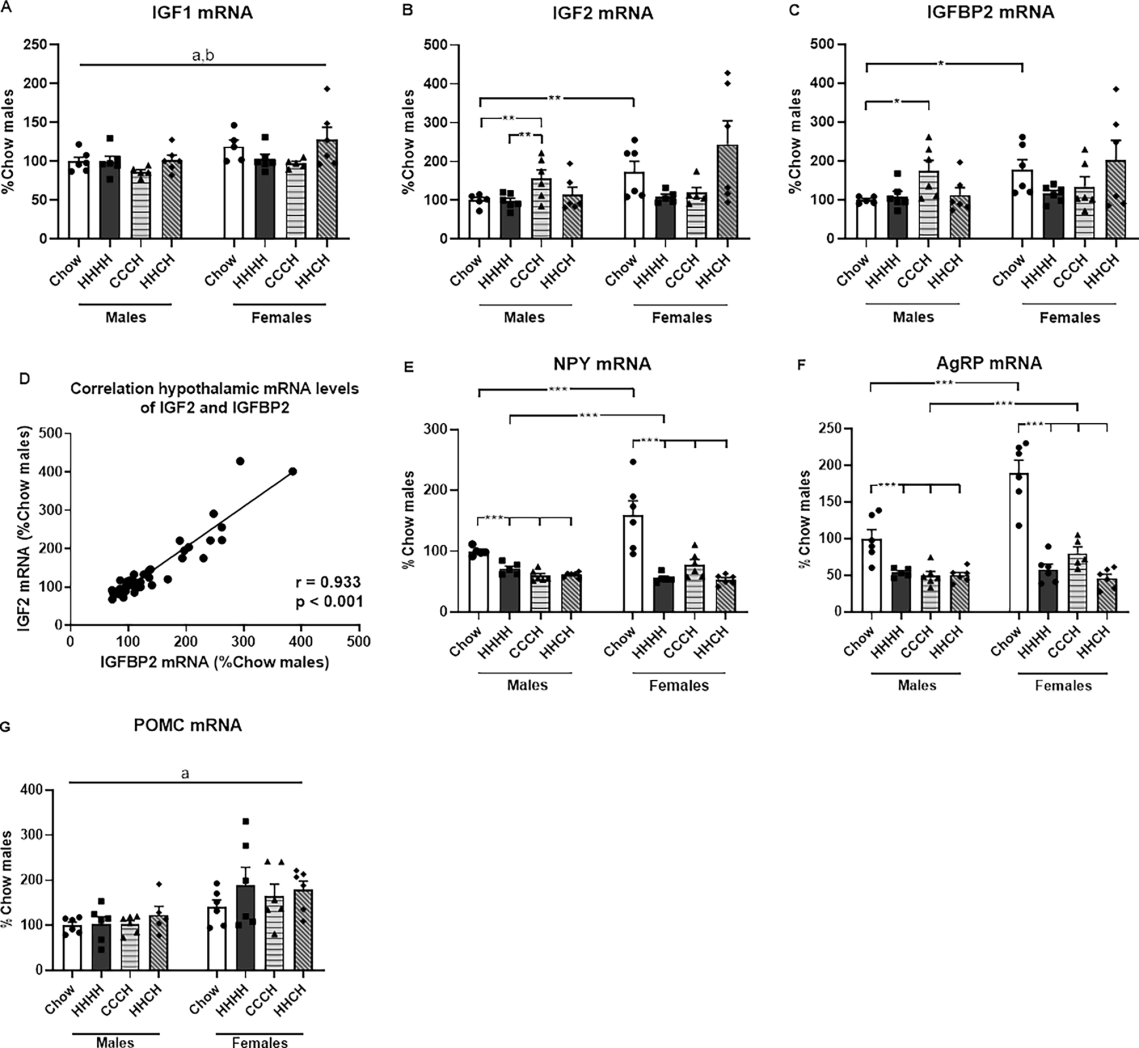
Relative mRNA levels of insulin-like growth factor (IGF) 1 (**A**), IGF2 (**B**), IGF binding protein (IGFBP)2 (**C**), neuropeptide Y (NPY; **E**), Agouti-related protein (AgRP; **F**), and proopiomelanocortin (POMC; **G**) in the hypothalamus, as well as the correlation of hypothalamic IGF2 and IGFBP2 mRNA levels (**D**), in mice fed chow or a high-fat diet (HFD) for 4 months (HHHH), a chow diet for 3 months and an additional month on a HFD (CCCH), or HFD for 2 months followed by chow for 1 month and then HFD for the last month (HHCH). *p < 0.05, ***p < 0.001. a: effect of the sex, b: effect of the diet. n = 6.

Relative hypothalamic IGF1 mRNA levels (Fig. 6A, statistics Supplementary Table 5) were higher in females than males and decreased in CCCH mice of both sexes. IGF2 mRNA levels (Fig. 6B) were also higher in females than males on a chow diet (F_(1,10)_ = 5.9, p < 0.05). The response to the diet differed between the sexes with male CCCH mice having higher IGF2 mRNA levels than chow and HHHH mice (F_(3,22)_ = 3.0, p = 0.05). A similar effect was found on IGFBP2 mRNA levels (Fig. 6C), with chow females having higher IGFBP2 mRNA levels than males (F_(1,10)_ = 7.4, p < 0.05) and with this binding protein increasing in CCCH males (F_(3,22)_ = 3.3, p < 0.05). As previously shown^24,37,39^, relative hypothalamic mRNA levels of IGF2 and IGFBP2 were positively correlated (r = 0.933, p < 0.001; Fig. 6D).

### Hypothalamic metabolic neuropeptides

Hypothalamic NPY mRNA levels (Fig. 6E, statistics Supplementary Table 5) were influenced by sex and diet, with an interaction between these factors. Females had higher NPY mRNA levels than males when on a chow diet (F_(1,10)_ = 5.5, p < 0.05), but lower levels than males in the HHHH (F_(1,9)_ = 7.8, p < 0.05) and HHCH (F_(1,11)_ = 4.8, p = 0.05) groups. Decreased NPY mRNA levels were found in the HHHH, CCCH and HHCH groups in both males (F_(3,21)_ = 30.6, p < 0.001) and females (F_(3,22)_ = 15.0, p < 0.001). Relative AgRP mRNA levels (Fig. 6F) were higher in females than in males on chow (F_(1,11)_ = 17.3, p < 0.01), as well as on the CCCH dietary regimen (F_(1,10)_ = 8.9, p < 0.05). The HHHH, CCCH and HHCH groups had lower AgRP mRNA levels than chow-fed mice in both males (F_(3,22)_ = 11.2, p < 0.001) and females (F_(3,22)_ = 36.2, p < 0.001). Females had overall higher POMC mRNA levels (Fig. 6G) than males, with no effect of diet in either sex.

### IGF2 effects in astrocyte cultures

**Figure 7:**
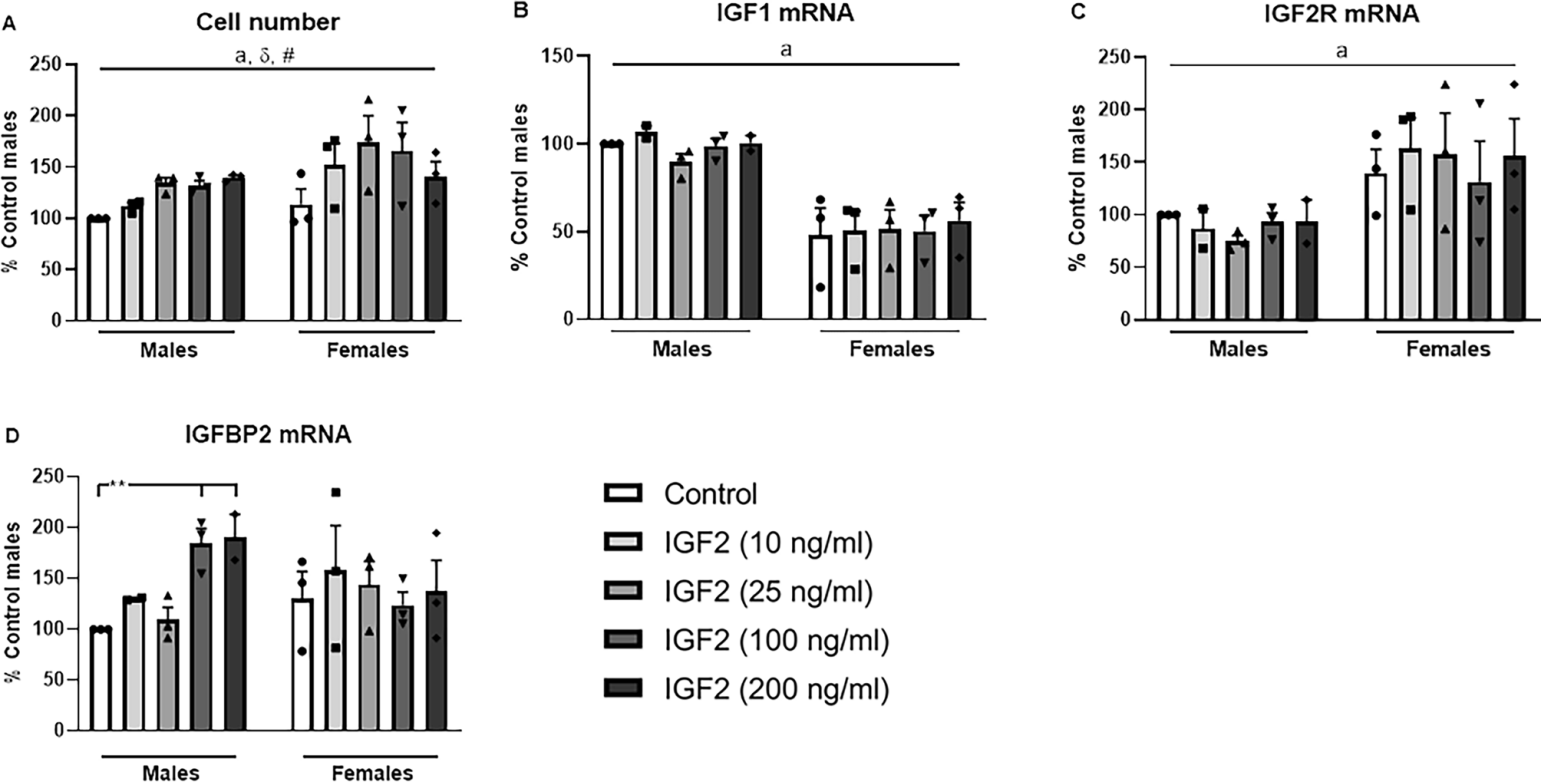
Cell number (**A**) and the relative IGF1 (**B**), IGF2 receptor (IGF2R, **C**) and IGFBP2 (**D**) mRNA levels in hypothalamic astrocyte cultures from male and female rats after 24 h of IGF2 treatment at 10, 25, 100 or 200 ng/ml. **p < 0.01. a: effect of the sex; #, general effect of IGF2; δ, effect of the dose of IGF2. n = 3.

As a positive correlation between hypothalamic IGF2 and IGFBP2 were found here and previously^24,37,39^, we analyzed whether hypothalamic astrocytes produce IGFBP2 in response to IGF2. As IGFs are mitogenic^52–55^, we analyzed cell number (Fig. 7A), which was affected by sex, with a general effect of IGF2 and the IGF2 dose (statistical analysis can be found in Supplementary Table 6). Indeed, IGF2 increased cell number in both sexes. IGF2 treatment did not alter relative IGF1 mRNA levels (Fig. 7B), nor those of IGF2R (Fig. 7C), but there was an effect of sex in both cases. In male astrocytes, IGF2 increased IGFBP2 mRNA levels (Fig. 7D) at higher concentrations (F_(1,12)_ = 4.5, p < 0.05).

## Discussion

Here we show that not only are there sex differences in weight gain, energy intake and energy efficiency after HFD intake, as previously reported^28,29,56–60^, as well as in weight loss in response to a healthy diet^61,62^, but that the response to resumption of a HFD after weight loss on an unrestricted healthy diet also differs between the sexes. Indeed, laboratory chow diets have been developed as a healthy nutritional choice for rodents, due to its energy content, the low amount of fats (especially saturated fats), the inclusion of complex carbohydrates and fiber, as well as the required vitamins and minerals^63^. A main difference between the sexes is that males modulate their Kcal intake on a HFD, returning to control levels, while females continue to have an elevated energy intake. Although both sexes drastically reduced energy intake when returned to the healthier chow diet after consuming HFD, overweight females required more time to achieve significant weight loss and lost less weight than males. Dissimilarities were also found in insulin resistance, with males being more affected as previously reported^64^, as well as in the central IGF system, which was also modulated in males. These results are in agreement with the observations that women are reported to be more efficient in defending body weight and glucose parameters than men^65^. Indeed, the slower changes in body weight in females after both increased and decreased caloric intake could involve the protective role of estrogens^32,66^.

As previously shown, on a HFD females consumed more kilocalories per gram of body weight compared to males^59^ and males had a higher energy efficiency^57^ and gained weight more rapidly. Diets rich in fat are more palatable than chow, and female rats consume palatable foods independently of their satiety state^67^, while males more readily adjust their energy consumption to their hunger state; however, both males and females are reported to eat standard chow according to their hunger state^68^. The difference in palatability of the diets could also explain the drastic reduction in energy intake when the mice were given chow after HFD.

Sex differences in the hypothalamic circuitry that regulates homeostatic food intake and energy expenditure^69^ most likely underlie some of the metabolic dissimilarities between males and females observed here. Male mice re-exposed to HFD and those given HFD for the first time initially consumed more kilocalories than HHHH mice. This was not observed in females. It is possible that in females effects on food intake were not found due to the high variability in intake over time, especially in the HFD-exposed mice and differences in the estrous cycle stage could be involved in this variability. Indeed, female mice were only synchronized and the estrous stage analyzed at the end of the study; thus, females could be at one of the four different stages of the estrous cycles, which are known to differentially affect to food intake^70^.

Despite some sex differences, mice gained and lost weight after the dietary changes with no difficulty and with no apparent difference on weight gain between mice re-exposed or exposed to HFD for the first time. Likewise, the percentage of adipose tissue and circulating leptin levels increased with HFD in both sexes, regardless of the HFD regimen. One might speculate that some effects of HFD intake might require more time, with a greater weight gain and longer development of secondary complications required to cause more long-lasting and profound effects on the metabolic control system such that it is not totally normalized with weight loss. Indeed, the time an individual is obese is important for the rebound effect on adiposity in humans^71^. However, we observed no rebound effect, which could be because here mice were never on a restrictive diet, which can alter hedonic behaviors that can influence ingestion when exposed to palatable foods; this may also underlie some differences with other interventional studies.

Alterations in glucose metabolism were observed in response to long-term HFD intake, as previously reported^59,60,72^, with no differences in glycemia or the AUC being observed between the HHHH, CCCH and HHCH groups. However, male HHCH mice had higher insulin and HOMA-IR than HHHH mice. This indicates that glucose metabolism was not totally normalized with weight loss and/or it became more susceptible to further dietary assaults, as HHCH male mice required higher levels of insulin to maintain normal glycemia. In females no differences were found between HHHH and HHCH mice groups. Affectation of glucose tolerance occurred as early as one month after HFD intake in male mice, while females ingesting a HFD for only one month had lower insulin and HOMA levels compared to both HHHH continuous-HFD intake and HHCH mice, again indicating that females are more resistant to these modifications. Basal glycemia at sacrifice was not affected by diet, whereas basal glycemia in the GTT was higher in the groups that consumed HFD, which might be explained by the differences in the duration of fasting (12 h before sacrifices and 6 h before GTT) may explain this discrepancy.

Sex differences in the circulating IGF system have been reported in humans^35,36,73^ and rodents^25,74^. Here we observed higher peripheral levels of IGF2 in females compared to males, but no sex differences in total or free IGF1 levels, as previously reported^74^. This is in agreement, some studies showing no sex differences in total IGF1^25^ or free IGF1 levels through development^74^, but not with others^24,39^. Circulating total IGF1 levels increased in mice of both sexes with HFD-induced weight gain, as in previous studies^75,76^; however, no dietary changes in free IGF1, IGF2 or IGFBP2 levels were found here. Some studies report an increase^38,77^ and others a reduction^78^ in circulating IGF2 levels in patients with obesity, with these differences possibly being due to the degree and/or duration of obesity. IGFBP2, with reduced circulating levels in patients with obesity^34,38,79^ and animal models of obesity^80^, is purported to be protective against obesity and diabetes onset^42,43^. However, no changes in circulating IGFBP2 levels were observed here. This apparent discrepancy with our previous studies could be associated with the lack of dietary effect on basal glycemia in the study presented here.

Increased central IGF1 improves peripheral glucose metabolism and increases insulin sensitivity^44^. Moreover, increased hypothalamic IGF1 levels could be involved in protection against HFD-induced metabolic damage. Hypothalamic IGF1 mRNA levels increased in male mice after 7 weeks of HFD intake^81^, but here they were reduced after 4 weeks (CCCH) in both sexes, while after 4 months of HFD no changes were found. This indicates dynamic changes in this growth factor in response to metabolic modifications that deserve further investigation. Elevated circulating IGF2 is reported to exert a negative feedback effect on IGF1 levels^82^ and the reduction in IGF1 mRNA levels in CCCH mice could be a consequence, at least in part, of increased IGF2 production. However, when we analyzed whether astrocytes, one of the main sources of these factors centrally, might be involved in this control, we saw no effect of IGF2 on IGF1 mRNA levels, suggesting that other signals and/or cell types are involved.

Hypothalamic IGF2 mRNA levels were increased in CCCH males, with no changes found in females. However, hypothalamic IGF2 mRNA was previously reported to increase after 2 months of HFD intake in females^37^. This differential timing in IGF2 modifications could be associated with differences between the sexes in the onset of alterations in glucose metabolism^83^. In agreement, both glycemia and the hypothalamic IGF2 mRNA levels increase after more extended exposure to HFD in females^37^. IGFBP2 mRNA levels were also increased exclusively in the hypothalamus of male CCCH mice. As IGFBP2 is reported to exert a protective role against obesity and diabetes onset^42,43^, and glucose metabolism is more rapidly affected in males after HFD intake^57,72,84^, one might speculate that the novel HFD exposure results in activation of protective mechanisms. Hypothalamic IGF2 and IGFBP2 mRNA levels were positively correlated, as reported previously^24,37,39^. In male astrocytes, IGF2 increased IGFBP2 mRNA levels suggesting that these glial cells might be involved in the central association of these factors. This binding protein has a higher preference for binding IGF2 over that of IGF1^85^ and may act as a regulator of this ligand^86^. Thus, the increase in IGFBP2 mRNA could be to regulate IGF2 levels^87^.

Females had higher mRNA levels of NPY, AgRP and POMC than males, as previously reported^88,89^. The levels of NPY and AgRP mRNA were decreased as early as one month after HFD intake in both sexes, a previously reported phenomenon interpreted as homeostatic response to reduce food intake^90,91^ and indeed, a reduction in energy intake was observed after the initial hyperphagic period, especially in males. Other studies indicate no change in these orexigenic neuropeptides after two weeks of HFD in male mice^92^ or after longer periods (4 to 8 weeks)^93^. We previously reported that expression of both orexigenic neuropeptides returned to control levels after one month of a chow diet and weight loss in both sexes^39^, indicating that the plasticity of this system in response to diet changes and weight loss was maintained.

No effects of diet on hypothalamic POMC mRNA levels were observed here. Hypothalamic POMC mRNA levels have been reported to increase in male mice after 7^81^ and 11 weeks of HFD^94^, or to not change in male mice after 16 weeks^95^ or in female mice after 20 weeks^96^ of HFD. The composition of the HFD could explain part of these differences.

In conclusion, males and females have different metabolic responses and the results reported here reinforce this concept, demonstrating dissimilarities in the response to different dietary changes. The weight gain in response to the re-exposition to HFD did not differ from that observed in mice ingesting HFD for the first time, indicating that greater weight gain and/or a longer period of being overweight may be necessary to observe a difference. However, it is also possible that the employment of a healthy diet to lose weight instead of food restriction underlies this observation and this deserves further investigation as it could be important for weight loss counseling. Moreover, we might speculate that following a restricted diet instead would lower resting metabolic rate and return to HFD would result in a rebound effect. Glucose metabolism was more affected by reestablishment of poor dietary habits in males, again demonstrating sex differences in this parameter, with the IGF system possibly being involved in this process, as it was differentially modulated in males and females and this clearly deserves further studies.

## Methods

### Ethical statement

This study was performed in accordance with the European Communities Council Directive (2010/63/UE) and the Royal Decree 53/2013 pertaining to the protection of experimental animals. It was also approved by the Ethical Committee of Animal Experimentation of the Hospital Puerta de Hierro of Madrid and the Animal Welfare Organ of the Community of Madrid. Mice were maintained at 22 ± 2 °C with free access to tap water in a stable 12-h light–dark cycle. The study design and reporting of the results adheres to the ARRIVE guidelines (https://arriveguidelines.org).

### Animals and diets

Seventy-two six-week-old C57BL/6J mice (36 males and 36 females) were purchased from Charles River Laboratories (Sant Cugat del Vallès, Barcelona, Spain). Upon their arrival, animals were weighed, and 3 mice of the same sex were randomly placed in each cage. They had free access to standard rodent chow and tap water while being allowed to acclimate to their new environment for one week.

For the study, one group (Chow) of mice was fed a standard rodent chow diet (6% Kcal from fat, 17% Kcal from proteins, 77% from carbohydrates, 3.41 kcal/g, Panlab, Barcelona, Spain) during the 4 months and served as our controls. Another group (HHHH) received a HFD (62% Kcal from fat, 18% Kcal from proteins, 20% Kcal from carbohydrates, 5.1 kcal/g, LabDiet, Sodispan Research SL, Madrid, Spain) for the same period of time. A third group received chow for 3 months and then was given the HFD for the final month (CCCH). To determine the effect of “re-exposition to HFD” mice were fed with HFD for the two initial months, switched to chow for the third month, and then was re-exposed to HFD for the last month of the study (HHCH). Nine animals of each sex were included in each group (n = 9), resulting in 3 cages per sex and diet. Body weight and food intake were monitored weekly until the termination of the study. Food intake was monitored by weighing the amount of food remaining of a previously determined quantity, with the pieces that fell into the cages being taken into account. However, a caveat that should be taken into account is that due to the lower consistency of the HFD, it breaks more easily and the recovery may be less efficient. Mean energy efficiency during the entire study was calculated as the weight gained in grams per the total number of calories consumed.

### Glucose tolerance test (GTT)

One week prior to the end of the study, a glucose tolerance test (GTT) was performed as described^37,39^. Six animals per group were randomly chosen and then fasted for 6 h but maintaining free access to tap water. Mice were weighed and intraperitoneally injected with a D-glucose solution in PBS (0.4 g/ml) at a dose of 2 mg per gram of body weight. A Freestyle Optimum Neo glucometer (Abbott, Whitney, UK) was employed for determining glycemia at 0 (basal), 30-, 60-, 90-, and 120-min post injection.

### Sacrifices and tissue collection

To synchronize the estrous cycle in female mice thus avoiding additional variability, bedding from male cages was mixed in the bedding of the females’ cages 3–4 days before sacrifice. The estrous cycle stage was determined by vaginal cytology at sacrifice, with 83% of the females being sacrificed at estrous. Mice were weighed and fasted 12 h prior to sacrifice, which took place by decapitation after rapid exposure to CO_2_ to reduce suffering between 09:00 and 11:00 am. Glycemia levels were measured with a Freestyle Optimum Neo glucometer (Abbott). Peripheral blood was collected in tubes with a 0.5 M ethylenediaminetetraacetic acid (EDTA) solution to prevent clotting. Tubes were centrifuged at 3000 rpm for 15 min at 4 °C and plasma was aliquoted and maintained at − 80 °C.

In mice, the brain was extracted from skull after decapitation, and the hypothalamus (rostrally limited by the optic chiasm and caudally by the anterior margin of the mammillary bodies) was then dissected and frozen. Retroperitoneal visceral adipose tissue and inguinal subcutaneous white adipose tissue depots were dissected, weighed and frozen. All tissues were stored at − 80 °C until processing.

### Enzyme-linked immunosorbent assays (ELISAs)

Plasma levels of free IGF1 (Ref.: AL-136, AnshLabs, Webster, TX, USA), total IGF1 (Ref.: E25; Mediagnost, Reutlingen, Germany), IGF2 (Ref.: MG200; R&D Systems, Minneapolis, Minnesota, USA), IGFBP2 (Ref.: RAB0234; Millipore, Burlington, MA, USA), insulin (Ref.: EZRMI-13K; Millipore) and leptin (Ref.: EZML-82K; Millipore) were assayed according to manufacturer’s instructions, and absorbance was read using a TECAN Infinite M200. Homeostatic Model Assessment for Insulin Resistance (HOMA-IR) was calculated according to the equation:

$$HOMA-IR=\frac{glycemia \left(\frac{mmol}{l}\right)\times insulin (\frac{mU}{l})}{22.5}$$

### Quantitative real-time polymerase chain reaction (RT-qPCR)

An RNeasy Plus Mini Kit (Qiagen, Hilden, Germany) was used to isolate RNA according to the manufacturer’s instructions. The RT-PCR assays were performed as previously described^57^, in which 0.5 μg of RNA were used and the NZY First-Strand cDNA Synthesis Kit (NZYTech, Lisbon, Portugal) was used for retrotranscription. TaqMan probes for the genes of interest (Table 2) were employed, and a QuantStudio 3 Real-Time PCR System (Applied Biosystems, Carlsbad, CA, USA) was used for signal detection.

**Table 2:** List of TaqMan probes used for qPCR.

Name	Gene	Reference (rat)	Reference (mouse)
Agouti-related protein	*Agrp*	N/A	Mm00475829_g1
Insulin-like growth factor 1	*Igf1*	Rn99999087_m1	Mm00439560_m1
Insulin-like growth factor 2	*Igf2*	Rn01454518_m1	Mm00439564_m1
Insulin-like growth factor 2 receptor	*Igf2r*	Rn01636937_m1	N/A
Insulin-like growth factor-binding protein 2	*Igfbp2*	Rn00565473_m1	Mm00492632_m1
Neuropeptide Y	*Npy*	N/A	Mm03048253_m1
Pro-opiomelanocortin	*Pomc*	N/A	Mm00435874_m1

As the housekeeping gene, an endogenous GAPDH control (Applied Biosystems) was used, and the ΔΔCt method was applied for the analyses. In the in vivo studies, the results were normalized and expressed in percentage in comparison with the male chow group, whereas in the in vitro studies the reference group was the control treatment in astrocytes from male rats.

### Primary hypothalamic astrocyte cultures

Primary astrocyte cultures were obtained from PND2 Wistar rat pups and performed as previously described^97^. Male and female pups were differentiated by ano-genital distance and cultured separately. Rat pups were sacrificed by decapitation, the brains extracted and the hypothalami dissected, with the meninges being carefully discarded. Astrocyte cultures were maintained in DMEM/F12 media (Gibco, Invitrogen Co., Thermo-Fisher, Waltham, Massachusetts, USA) enriched with 10% fetal bovine serum (FBS, Sigma-Merck, Darmstadt, Germany) and treated with 1% penicillin/streptomycin and anti-mycotic (Gibco), with the medium being changed every 2–3 days. When the cell confluence was approximately 70%, the flasks were placed in an Ecotron orbital shaking incubator for 16 h at 280 rpm and 37 °C to eliminate remaining oligodendrocytes and microglia. Cells were then trypsinized and counted by using a Countess II FL Automated Cell Counter (Thermo-Fisher) and seeded at 1.5 × 10^4^ cells/cm^2^ in 100 mm culture plates and allowed to recover for 24 h. Cells were serum-starved for 24 h prior to the treatments. For the different experiments, astrocytes were treated for 24 h with an IGF2 (I8904, Sigma-Merck) solution in PBS at 10, 25, 100 or 200 ng/ml supplemented with 0.1% BSA, as described^98^, or with vehicle.

#### Cell number estimation by crystal violet staining

Cells were seeded at 3.5 × 10^4^ cells per well in 24-well plates for 24 h. After serum-starvation as described above, treatments were added for 24 h. After treatment, a 1% glutaraldehyde solution in PBS was added for 15 min, rinsed and then a 0.1% crystal violet (Sigma-Merck) solution was added for 20 min and then thoroughly rinsed. When the plates were dry, a 10% acetic acid solution was added, and the dye solubilized for 5 min. The samples were added to a 96 well plate and the absorbance at 590 nm was read on a TECAN Infinite M200. The absorbance in each well was normalized to the control values.

### Statistical analyses

Statistical analyses were performed with SPSS 15.0 (SPSS Inc., Chicago, IL, USA) software, whereas graphs were made with GraphPad Prism 8 software (San Diego, CA, USA). In the in vivo studies, a 2-way ANOVA with the factors of sex and diet (chow, HHHH, CCCH or HHCH) was performed, which was followed by a 1-way ANOVA when appropriate. In all experiments, weight gain and food intake over time, as well as glycemia changes during the GTT, were analyzed by a 3-way ANOVA with repeated measures with time, sex and the diet as factors. In the in vitro study, a 2-way ANOVA was used to determine the effects and interaction between the sex and the IGF2 dose (0, 10, 25, 100 or 200 ng/ml). A 2-way ANOVA with sex and the presence of IGF2 regardless of dose was also calculated. A Pearson correlation coefficient was calculated for the linear correlation between variables. In all analyses, p < 0.05 was considered significant.

### Supplementary Information

Supplementary Information.

## Data Availability

The raw data supporting the conclusions of this article will be made available by the authors, without undue reservation by contacting Dr. J.A. Chowen.
