# Supplementary material for: The metabolic effects of resumption of a high fat diet after weight loss are sex dependent in mice
Source: Sci Rep. 2023 Aug 14;13:13227. doi: 10.1038/s41598-023-40514-w (PMC10425431; doi:10.1038/s41598-023-40514-w)
Supplement: Supplementary file 1 — Supplementary Information. [file 41598_2023_40514_MOESM1_ESM.pdf]

Supplementary Information

The metabolic effects of resumption of a high fat diet after weight loss are sex dependent in mice

Santiago Guerra-Cantera[1,2,3], Laura M. Frago[1,2,3], María Jiménez-Hernaiz[1,3], Roberto Collado-Pérez[1,2], Sandra Canelles[1,3], Purificación Ros[2,4], Jorge García-Piqueras[1], Iris Pérez-Nadador[1], Vicente Barrios[1,3], Jesús Argente[1,2,3,5*], Julie A. Chowen[1,3,5*]

[1] Department of Endocrinology, Hospital Infantil Universitario Niño Jesús, Instituto de Investigación La Princesa, Madrid, Spain.

[2] Department of Pediatrics, Universidad Autónoma de Madrid, Madrid, Spain.

[3] Centro de Investigación Biomédica en Red de Fisiopatología de la Obesidad y Nutrición (CIBEROBN), Instituto de Salud Carlos III, Madrid, Spain.

[4] Department of Endocrinology, Hospital Universitario Puerta de Hierro-Majadahonda, Madrid, Spain.

[5] IMDEA Food Institute, CEI UAM + CSIC, Madrid, Spain.

	Interaction			Independent effect		
	**Factors**	**F**	**p-value**	**Factor**	**F**	**p-value**
**Body weight (global)**	Sex-Time	$F_{(16,71)} = 10.3$	$p<0.001$	Sex	$F_{(1,71)} = 198.0$	$p<0.001$
	Diet-Time	$F_{(16,71)} = 39.5$	$p<0.001$	Diet	$F_{(3,71)} = 37.3$	$p<0.001$
	Sex-Diet	$F_{(3,71)} = 4.4$	$p<0.01$	Time	$F_{(16,71)} = 357.1$	$p<0.001$
	Sex-Diet-Time	$F_{(16,71)} = 5.1$	$p<0.001$			
**Body weight (study onset)**	NS			Sex	$F_{(1,71)} = 245.9$	$p<0.001$
**Body weight (week 1)**	Sex-Diet	$F_{(3,71)} = 3.7$	$p<0.05$	Sex	$F_{(1,71)} = 344.4$	$p<0.001$
				Diet	$F_{(3,71)} = 10.3$	$p<0.001$
**Body weight (week 2)**	Sex-Diet	$F_{(3,71)} = 9.7$	$p<0.001$	Sex	$F_{(1,71)} = 384.6$	$p<0.001$
				Diet	$F_{(3,71)} = 20.8$	$p<0.001$
**Body weight (week 3)**	Sex-Diet	$F_{(3,71)} = 15.1$	$p<0.001$	Sex	$F_{(1,71)} = 404.7$	$p<0.001$
				Diet	$F_{(3,71)} = 32.6$	$p<0.001$
**Body weight (week 4)**	Sex-Diet	$F_{(3,71)} = 10.2$	$p<0.001$	Sex	$F_{(1,71)} = 283.1$	$p<0.001$
				Diet	$F_{(3,71)} = 36.0$	$p<0.001$
**Body weight (week 5)**	Sex-Diet	$F_{(3,71)} = 12.1$	$p<0.001$	Sex	$F_{(1,71)} = 232.7$	$p<0.001$
				Diet	$F_{(3,71)} = 39.7$	$p<0.001$
**Body weight (week 6)**	Sex-Diet	$F_{(3,71)} = 9.9$	$p<0.001$	Sex	$F_{(1,71)} = 232.7$	$p<0.001$
				Diet	$F_{(3,71)} = 39.7$	$p<0.001$
**Body weight (week 7)**	Sex-Diet	$F_{(3,71)} = 10.7$	$p<0.001$	Sex	$F_{(1,71)} = 190.8$	$p<0.001$
				Diet	$F_{(3,71)} = 50.3$	$p<0.001$
**Body weight (week 8)**	Sex-Diet	$F_{(3,71)} = 9.9$	$p<0.001$	Sex	$F_{(1,71)} = 165.0$	$p<0.001$
				Diet	$F_{(3,71)} = 54.0$	$p<0.001$
**Body weight (week 9)**	Sex-Diet	$F_{(3,70)} = 5.0$	$p<0.01$	Sex	$F_{(1,71)} = 149.2$	$p<0.001$
				Diet	$F_{(3,71)} = 39.7$	$p<0.001$
**Body weight (week 10)**	Sex-Diet	$F_{(3,70)} = 3.5$	$p<0.05$	Sex	$F_{(1,70)} = 127.1$	$p<0.001$
				Diet	$F_{(3,70)} = 39.5$	$p<0.001$
**Body weight (week 11)**	Sex-Diet	$F_{(3,70)} = 3.4$	$p<0.05$	Sex	$F_{(1,70)} = 116.7$	$p<0.001$
				Diet	$F_{(3,70)} = 49.6$	$p<0.001$
**Body weight (week 12)**	NS			Sex	$F_{(1,70)} = 111.5$	$p<0.001$
				Diet	$F_{(3,70)} = 52.4$	$p<0.001$
**Body weight (week 13)**	NS			Sex	$F_{(1,70)} = 92.4$	$p<0.001$
				Diet	$F_{(3,70)} = 27.1$	$p<0.001$
**Body weight (week 14)**	NS			Sex	$F_{(1,70)} = 98.4$	$p<0.001$
				Diet	$F_{(3,70)} = 31.6$	$p<0.001$
**Body weight (week 15)**	NS			Sex	$F_{(1,70)} = 79.8$	$p<0.001$
				Diet	$F_{(3,70)} = 31.9$	$p<0.001$
**Body weight (week 16)**	NS			Sex	$F_{(1,70)} = 79.9$	$p<0.001$
				Diet	$F_{(3,70)} = 30.0$	$p<0.001$

Supplementary Table 1. Significant results of the two-way ANOVA between sex and diet, showing the value of F and the p-value of the body weight by week. In the case of Body weight through the study (global), a three-way ANOVA with repeated measures with sex, dietary regimen and time as factors was performed. NS: not significant.

	Interaction			Independent effect		
	**Factors**	**F**	**p-value**	**Factor**	**F**	**p-value**
**Weight gain (%) from baseline to the end of month 2**	Sex-Diet	$F_{(3,71)} = 10.4$	$p<0.001$	Sex	$F_{(1,71)} = 33.2$	$p<0.001$
				Diet	$F_{(3,71)} = 10.4$	$p<0.001$
**Weight gain (%) from the end of month 2 to the end of month 3**	Sex-Diet	$F_{(3,71)} = 10.8$	$p<0.001$	Sex	$F_{(1,71)} = 10.0$	$p<0.01$
				Diet	$F_{(3,71)} = 135.0$	$p<0.001$
**Weight gain (%) from baseline to the end of month 3**	NS			Sex	$F_{(1,71)} = 4.9$	$p<0.05$
				Diet	$F_{(3,71)} = 42.2$	$p<0.001$
**Weight gain (%) from the end of month 3 to the end of month 4**	Sex-Diet	$F_{(3,70)} = 2.8$	$p<0.05$	Diet	$F_{(3,70)} = 109.7$	$p<0.001$
**Total weight gain (%) from baseline to the end of the study**	NS			Sex	$F_{(1,70)} = 5.1$	$p<0.05$
				Diet	$F_{(3,70)} = 34.0$	$p<0.01$
**Body weight at sacrifice**	NS			Sex	$F_{(1,70)} = 70.0$	$p<0.001$
				Diet	$F_{(3,70)} = 30.9$	$p<0.001$
**Lumbar adipose tissue**	NS			Diet	$F_{(3,71)} = 27.4$	$p<0.001$
**Subcutaneous adipose tissue**	NS			Diet	$F_{(3,71)} = 31.2$	$p<0.001$

Supplementary Table 2. Significant results of the two-way ANOVA between sex and diet, showing the value of F and the p-value of the weight gain and body parameters. NS: not significant.

	Interaction			Independent effect		
	Factors	F	p-value	Factor	F	p-value
**Energy intake (global)**	Diet-Time	$F_{(15,23)} = 6.4$	p<0.001	Time	$F_{(15,23)} = 7.0$	p<0.001
**Energy intake (week 1)**	NS			Diet	$F_{(3,23)} = 9.0$	p=0.001
**Energy intake (week 2)**	NS			Diet	$F_{(3,23)} = 3.9$	p<0.05
**Energy intake (week 3)**	NS			Diet	$F_{(3,23)} = 3.9$	p<0.05
**Energy intake (week 4)**	NS			Diet	$F_{(3,23)} = 3.3$	p<0.05
**Energy intake (week 5)**	NS			NS		
**Energy intake (week 6)**	NS			Diet	$F_{(3,23)} = 3.7$	p<0.05
**Energy intake (week 7)**	NS			NS		
**Energy intake (week 8)**	NS			Diet	$F_{(3,23)} = 3.2$	p=0.05
**Energy intake (week 9)**	NS			Diet	$F_{(3,23)} = 5.1$	p<0.05
**Energy intake (week 10)**	NS			Diet	$F_{(3,23)} = 6.5$	p<0.01
**Energy intake (week 11)**	NS			Diet	$F_{(3,23)} = 4.8$	p<0.05
**Energy intake (week 12)**	NS			NS		
**Energy intake (week 13)**	NS			NS		
**Energy intake (week 14)**	NS			Diet	$F_{(3,23)} = 6.5$	p<0.01
**Energy intake (week 15)**	Sex-Diet	$F_{(3,23)} = 3.1$	p=0.05	Diet	$F_{(3,23)} = 4.8$	p<0.05
**Energy intake (week 16)**	NS			NS		
**Total Kcal/mouse/day**	NS			NS		
**Total Kcal/mouse/day/ 100 g**	NS			Sex	$F_{(1,23)} = 6.7$	p<0.05
**Kcal/mouse/day from baseline to the end of month 2**	NS			Diet	$F_{(3,23)} = 3.6$	p<0.05
**Kcal/mouse/day from the end of month 2 to the end of month 3**	NS			Diet	$F_{(3,23)} = 4.6$	p<0.05
**Kcal/mouse/day from baseline to the end of month 3**	NS			Diet	$F_{(3,23)} = 3.1$	p=0.05
**Kcal/mouse/day from the end of month 3 to the end of month 4**	NS			Diet	$F_{(3,23)} = 3.6$	p<0.05
**Total energy efficiency**	NS			Sex	$F_{(1,23)} = 11.9$	p<0.01
				Diet	$F_{(3,23)} = 16.1$	p<0.001

Supplementary Table 3. Significant results of the two-way ANOVA between age and sex, showing the value of F and the p-value of the energy intake by week and of the global energy intake parameters. In the case of energy intake through the study (global), a three-way ANOVA with repeated measures with sex, dietary regimen and time as factors was performed. NS: not significant.

	Interaction			Independent effect		
	Factors	F	p-value	Factor	F	p-value
**GTT (global)**	Sex-Time	$F_{(4,46)} = 5.3$	$p<0.001$	Sex	$F_{(1,46)} = 13.1$	$p<0.001$
	Diet-Time	$F_{(4,46)} = 4.8$	$p<0.001$	Diet	$F_{(3,46)} = 35.9$	$p<0.001$
	Sex-Diet	$F_{(3,46)} = 4.8$	$p<0.01$	Time	$F_{(4,46)} = 156.2$	$p<0.001$
**Glycemia GTT (Basal)**	Sex-Diet	$F_{(3,46)} = 4.1$	$p<0.05$	Sex	$F_{(1,46)} = 13.9$	$p=0.001$
				Diet	$F_{(3,46)} = 11.7$	$p<0.001$
**Glycemia GTT (30 minutes)**	NS			Diet	$F_{(3,46)} = 5.9$	$p<0.01$
**Glycemia GTT (60 minutes)**	NS			Sex	$F_{(1,46)} = 11.2$	$p<0.01$
				Diet	$F_{(3,46)} = 45.6$	$p<0.001$
**Glycemia GTT (90 minutes)**	Sex-Diet	$F_{(3,46)} = 3.2$	$p<0.05$	Sex	$F_{(1,46)} = 11.2$	$p<0.01$
				Diet	$F_{(3,46)} = 18.6$	$p<0.01$
**Glycemia GTT (120 minutes)**	Sex-Diet	$F_{(3,46)} = 4.4$	$p<0.01$	Sex	$F_{(1,46)} = 16.1$	$p<0.001$
				Diet	$F_{(3,46)} = 20.0$	$p<0.001$
**Area Under Curve (AUC)**	Sex-Diet	$F_{(3,46)} = 4.4$	$p<0.01$	Sex	$F_{(1,46)} = 10.9$	$p<0.01$
				Diet	$F_{(3,46)} = 34.6$	$p<0.001$
**Insulin**	NS			Sex	$F_{(1,69)} = 14.6$	$p<0.001$
				Diet	$F_{(3,69)} = 7.3$	$p<0.001$
**Glycemia at sacrifices**	NS			Sex	$F_{(1,71)} = 8.6$	$p<0.01$
**HOMA-IR**	NS			Sex	$F_{(1,70)} = 17.2$	$p<0.001$
				Diet	$F_{(3,70)} = 5.7$	$p<0.01$

Supplementary Table 4. Significant results of the two-way ANOVA between sex and diet, showing the value of F and the p-value of the levels of glycemia during the glucose tolerance test (GTT) basally and at 30-, 60-, 90- and 120-minutes post injection. The same analyses were performed for the area under the curve (AUC), circulating insulin levels, glycemia at sacrifices, and the homeostatic model assessment for insulin resistance (HOMA-IR). In the case of GTT (global), a three-way ANOVA with repeated measures with sex, dietary regimen and time as factors was performed. NS: not significant.

	Interaction			Independent effect		
	**Factors**	**F**	**p-value**	**Factor**	**F**	**p-value**
**Free IGF1**	NS			NS		
**Total IGF1**	NS			Diet	$F_{(3,71)} = 5.0$	p<0.01
**IGF2**	NS			Sex	$F_{(1,71)} = 43.3$	p<0.001
**IGFBP2**	NS			NS		
**Leptin**	Sex-Diet	$F_{(3,69)} = 2.8$	p<0.05	Sex	$F_{(1,69)} = 6.7$	p<0.05
				Diet	$F_{(3,69)} = 19.8$	p<0.001
**Hypothalamic IGF1 mRNA**	NS			Sex	$F_{(1,44)} = 6.8$	p<0.05
				Diet	$F_{(3,44)} = 3.0$	p<0.05
**Hypothalamic IGF2 mRNA**	Sex-Diet	$F_{(3,44)} = 3.4$	p<0.05	Sex	$F_{(1,44)} = 4.8$	p<0.05
**Hypothalamic IGFBP2 mRNA**	Sex-Diet	$F_{(3,46)} = 2.3$	p=0.05			
**Hypothalamic NPY mRNA**	Sex-Diet	$F_{(3,44)} = 5.9$	p<0.01	Sex	$F_{(1,44)} = 3.9$	p=0.05
				Diet	$F_{(3,44)} = 23.5$	p<0.001
**Hypothalamic AgRP mRNA**	Sex-Diet	$F_{(3,45)} = 10.3$	p<0.001	Sex	$F_{(1,45)} = 19.8$	p<0.001
				Diet	$F_{(3,45)} = 46.2$	p<0.001
**Hypothalamic POMC mRNA**	NS			Sex	$F_{(1,46)} = 16.4$	p<0.001

Supplementary Table 5. Significant results of the two-way ANOVA between sex and diet, showing the value of F and the p-value of the levels of free and circulating total insulin-like growth factor 1 (IGF1), IGF2, insulin-like growth factor-binding protein 2 (IGFBP2) and leptin levels; and hypothalamic mRNA levels of IGF1, IGF2, IGFBP2, neuropeptide Y (NPY), Agouti related peptide (AgRP) and proopiomelanocortin (POMC). NS: not significant.

	Interaction			Independent effect		
	**Factors**	**F**	**p-value**	**Factor**	**F**	**p-value**
**Cell number (IGF2 treatment)**	NS			Sex	$F_{(1,29)} = 6.8$	$p<0.05$
				IGF2	$F_{(1,29)} = 9.7$	$p<0.01$
				IGF2 dose	$F_{(4,29)} = 2.9$	$p<0.05$
**IGF1 mRNA (IGF2 treatment)**	NS			Sex	$F_{(1,27)} = 56.3$	$p<0.001$
**IGF2R mRNA (IGF2 treatment)**	NS			Sex	$F_{(1,29)} = 8.1$	$p<0.01$
**IGFBP2 (males, IGF2 treatment)**	NS			IGF2	$F_{(1,12)} = 4.5$	$p<0.05$

Supplementary Table 6. Significant results of the two-way ANOVA between sex and insulin-like growth factor (IGF)2 presence, and also between sex and IGF2 dose, showing the value of F and the p-value of the cell number, IGF1, IGF2 receptor (IGF2R), and IGF-binding protein 2 (IGFBP2) in primary hypothalamic astrocyte cultures. NS: not significant.
